# Immunoinformatic development of a multiepitope messenger RNA vaccine targeting lipoate protein ligase and dihydrolipoamide dehydrogenase proteins of *Mycoplasma bovis* in cattle

**DOI:** 10.14202/vetworld.2025.1675-1684

**Published:** 2025-06-19

**Authors:** Dhafer Rasheed Al-Fetly, Dhama Alsallami, Amjed Alsultan

**Affiliations:** 1 Department of Internal and Preventive Medicine, College of Veterinary Medicine, University of Al-Qadisiyah, Al-Diwaniyah, Iraq; 2 Department of Physiology, Pharmacology and Biochemistry, College of Veterinary Medicine, University of Al-Qadisiyah, Al-Diwaniyah, Iraq

**Keywords:** cattle, epitope prediction, immunoinformatic, messenger RNA vaccine, multiepitope vaccine, *Mycoplasma bovis*

## Abstract

**Background and Aim:**

*Mycoplasma bovis* is a significant pathogen in cattle, causing respiratory, reproductive, and mammary diseases, leading to substantial economic losses. Conventional control measures remain ineffective due to antimicrobial resistance and the absence of an approved vaccine. This study aimed to develop a multiepitope messenger RNA (mRNA)-based vaccine against *M. bovis* using immunoinformatic and molecular modeling approaches.

**Materials and Methods:**

Two conserved surface-exposed proteins – lipoate protein ligase (LplA) and dihydrolipoamide dehydrogenase (PdhD) – were selected as vaccine targets. T- and B-cell epitopes were predicted using Immune Epitope Database and evaluated for antigenicity, allergenicity, toxicity, and conservancy. Selected epitopes were linked using specific amino acid linkers and combined with a resuscitation-promoting factor E (RpfE) adjuvant and untranslated regions (hemoglobin subunit beta and rabbit beta-globin) to improve translation and stability. The vaccine construct was modeled and validated through physicochemical profiling, secondary and tertiary structure prediction, molecular-docking with bovine toll-like receptors 4 (TLR4), and codon optimization. Molecular dynamics simulations were conducted to assess the stability of the vaccine-receptor complex.

**Results:**

The modeled vaccine construct contained five cytotoxic T lymphocyte, six helper T lymphocyte, and five B-cell epitopes. The construct was predicted to be highly antigenic (score: 0.835), non-allergenic, and non-toxic. Structural validation showed 93.5% of residues in favored regions of the Ramachandran plot and a Z-score of −10.6. Docking simulations revealed strong binding affinity to bovine TLR4, supported by robust molecular dynamics simulation outcomes, including high stability, low eigenvalues, and favorable covariance patterns. Codon optimization yielded a guanine-cytosine content of 59.8% and a codon adaptation index of 0.87, indicating efficient expression in cattle. The predicted mRNA structure exhibited good thermodynamic stability (minimum free energy: −321.42 kcal/mol).

**Conclusion:**

This study presents a computationally designed mRNA vaccine candidate against *M. bovis* based on LplA and PdhD epitopes. The construct demonstrated promising immunogenicity, structural integrity, and receptor-binding properties, representing a viable vaccine strategy. Nonetheless, *in vitro* and *in vivo* validation is essential to confirm the construct’s efficacy and safety in cattle.

## INTRODUCTION

*Mycoplasma* species are diminutive, self-replicating microorganisms with genome sizes ranging from 500 kb to 1500 kb. These organisms are capable of infecting a wide variety of hosts, including mammals, birds, reptiles, insects, and plants [1, 2]. They are characterized by distinctive features, such as reduced genome size, absence of biosynthetic pathways, low guanine-cytosine (GC) content, and the unconventional use of the UGA codon to encode tryptophan, setting them apart from other bacterial taxa [3]. Infections attributed to *Mycoplasma* result in notable economic burdens, primarily due to diagnostic efforts, therapeutic interventions, and decreased productivity in livestock [3, 4].

*Mycoplasma* bovis, a member of the class *Mollicutes*, is a significant pathogen in cattle, causing respiratory illness, mastitis, and reproductive abnormalities of varying severity [5, 6]. This pathogen was initially isolated from pneumonic cattle in 1960.

The international spread of *M. bovis* has been linked to the movement of animals across borders [7]. Transmission can occur through several routes, including maternal contact, contaminated milk, nasal discharges, and urine from infected individuals [8, 9]. The bacterium can affect cattle of all ages and adheres efficiently to host tissues due to its lack of a cell wall [3, 9]. Its incomplete biosynthetic machinery relies on glycerol as a primary energy source. *M. bovis* encodes roughly 47 lipoproteins, 12 extracellular proteins, and 18 transmembrane pro-teins, whose variability facilitates immune evasion. Genomic analyses have delineated two major groups of virulence-associated genes in *M. bovis* [10]. Several lipoproteins act as virulence determinants, with their genes often co-localized with ATP-binding cassette transporter operons [11–13]. Among these, lipoate protein ligase (LplA) and dihydrolipoamide dehydrogenase (PdhD) proteins have emerged as promising vaccine targets due to their crucial involvement in bacterial metabolism and interactions with the host. LplA plays a role in lipoic acid meta-bolism, essential for bacterial energy generation and persistence, while PdhD functions within the pyruvate dehydrogenase complex, a key element of central metabolic processes. These proteins are conserved, surface-accessible, and immunogenic, making them suitable for eliciting broad, protective immune responses across diverse *M. bovis* strains [14, 15].

Vaccination remains one of the most feasible and proactive measures to prevent *M. bovis* infections, particularly considering the limitations posed by antimicrobial resistance and suboptimal biosecurity practices. Computational (*in silico*) approaches provide an efficient framework for rational vaccine design, enabling expedited and cost-effective evaluation of candidate efficacy [16, 17]. Furthermore, molecular docking techniques assist in predicting the interaction between designed vaccines and host immune receptors [18, 19]. Multiepitope-based vaccines offer a compelling immunization strategy against *Mycoplasma* species, as they are highly specific, stable, and amenable to scalable production and delivery systems [20, 21]. The advancement of vaccine design against *M. bovis* has been significantly enhanced by immunoinformatics, which enables precise identification and assessment of antigenic epitopes using protein database resources [22].

Despite the increasing incidence and global spread of *M. bovis* infections in cattle, there is currently no commercially licensed vaccine that provides effective and broad-spectrum protection. Conventional control strategies, including antimicro-bial treatment and biosecurity measures, have proven inadequate due to the pathogen’s inherent resis-tance mechanisms, immune evasion strategies, and widespread transmission routes. Existing experimental vaccines, primarily based on whole-cell or inactivated preparations, have demonstrated limited efficacy and inconsistent immunogenic responses. Moreover, the high antigenic variability among *M. bovis* strains poses a significant obstacle to the development of universal vaccines. Although several immunoinformatic approaches have been explored for other pathogens, relatively few studies have harnessed reverse vaccinology or mRNA-based platforms to develop multiepitope subunit vaccines specifically targeting conserved and functionally essential proteins of *M. bovis*. In addition, while LplA and PdhD proteins are recognized for their metabolic and virulence-associated roles, their utility as antigenic targets for mRNA-based vaccine constructs remains underexplored in the scientific literature. This underscores the need for computationally driven vaccine development strate-gies that integrate epitope prediction, immunogenic profiling, molecular docking, and mRNA stabilization to generate safe, stable, and highly immunogenic vaccine candidates against *M. bovis*.

The primary aim of this study was to design and computationally evaluate a multiepitope mRNA-based vaccine targeting *M. bovis* infection in cattle using immunoinformatics and structural modeling tools. Specifically, the study focused on identifying and selecting antigenic, conserved, non-toxic, and non-allergenic epitopes from two essential surface-exposed proteins – LplA and PdhD. These epitopes were assembled into a single chimeric mRNA construct incorporating a molecular adjuvant and untranslated regions to enhance antigen presentation and mRNA stability. Comprehensive *in silico* analyses were performed to assess the physicochemical properties, secondary and tertiary structure, immunogenicity, receptor binding affinity (through docking with bovine toll-like receptors [TLR4]), and codon optimization of the vaccine. Ultimately, this study aims to provide a rationally designed, novel mRNA vaccine construct that can serve as a promising candidate for further experimental validation and eventual application in controlling *M. bovis* infections in cattle.

## MATERIALS AND METHODS

### Ethical approval

This study is computer based and does not involve the use of any animal or human samples.

### Study period and location

The study was conducted from January to November 2024 at the College of Veterinary Medicine, University of Al-Qadisiyah.

### Retrieval of LplA and PdhD protein sequences

The amino acid (AA) sequences of *M. bovis* Hubei-1 proteins – LplA (UniProt accession: A0A059Y7J7) and PdhD (UniProt accession: A0A1B0Z6M0) – were retrieved from the UniProt Knowledgebase. These proteins are either secreted or located on the bacterial surface, making them accessible to the host’s immune system. Their selection as vaccine antigens was based on the premise that targeting surface-exposed proteins can elicit robust immune responses, thereby interfering with bacterial metabolism and facilitating pathogen clearance [23].

### Epitope prediction and selection

Cytotoxic T lymphocyte (CTL), helper T lymphocyte (HTL), linear B-cell, and conformational B-cell epitopes were predicted from conserved regions of LplA and PdhD using the Immune Epitope Database (IEDB) web server (the National Institute of Allergy and Infectious Diseases, USA) [24]. To exclude cross-reactivity with host proteins, the predicted epitopes were subjected to homology analysis against all Bovidae peptides (taxid: 9895) using National Center for Biotechnology Information Basic Local Alignment Search Tool. The conservancy of each epitope was analyzed using the IEDB conservancy tool [25], while antigenicity was assessed using VaxiJen v2.0 (Edward Jenner Institute) [26]. Allergenicity predictions were performed with the AllerTOP server [27].

### Construction of the mRNA-based multiepitope vaccine

Selected epitopes were assembled into a single construct using appropriate AA linkers: GPGPG for CTL epitopes, AAY for HTL epitopes, and KK for B-cell epitopes. To enhance immunogenicity, the RpfE protein from *Mycobacterium tuberculosis* (UniProt ID: O53177) was included at the N-terminal as a built-in adjuvant. Hemoglobin subunit beta (HBB) and rabbit beta-globin (Rabb) sequences were fused as untranslated regions to both ends of the construct to enhance mRNA stability. In addition, a Kozak sequence and a signal peptide were incorporated at the N-terminal to facilitate translation and intracellular trafficking.

### Immunological and physicochemical profiling

The antigenicity and allergenicity of the vaccine construct were evaluated using VaxiJen v2.0 (//www.ddg-pharmfac.net/vaxijen) and AllerTOP (//www.ddg-pharmfac.net/allertop) respectively. Toxicity was predicted through the ToxinPred tool. Physicochemical properties, including molecular weight, theoretical isoelectric point, hydropathicity, instability index, and half-life, were analyzed using the ProtParam server (//web.expasy.org/protparam/) [28].

### Secondary structure prediction

The secondary structural elements of the vaccine protein – alpha helices, beta strands, and random coils – were predicted using the Prabi server (//doua.prabi.fr/) [29], providing insights into structural composition and potential epitope exposure.

### Tertiary structure modeling, refinement, and validation

The three-dimensional structure of the vaccine construct was predicted using the trRosetta server (//yanglab.qd.sdu.edu.cn/trRosetta/). Structural refinement was carried out using the Galaxy-Refine tool (//galaxy.seoklab.org/refine/), and validation was conducted thro-ugh the SAVES v6.1 platform (//saves.mbi.ucla.edu/). Structural accuracy was confirmed through Ramachandran plot analysis (//prosa.services.came.sbg.ac.at/prosa.php) and ProSA Z-score calculations [30, 31].

### Molecular docking with bovine TLR4

To assess the potential interaction between the vaccine and host immune receptors, molecular docking was performed using ClusPro v2.0 (//cluspro.org/) [31]. The structure of bovine TLR4 (UniProt accession: Q9GL65) was retrieved from UniProt. The refined vaccine model with the highest confidence score was selected for docking simulations.

### Codon optimization and mRNA secondary structure

Codon optimization of the vaccine nucleotide sequence was conducted using the VectorBuilder tool, which provided GC content and codon adaptation index (CAI) values to predict expression efficiency in bovine cells. The secondary structure and minimum free energy of the mRNA transcript were predicted using RNAfold (//rna.tbi.univie.ac.at/cgi-bin/RNAWebSuite/RNAfold.cgi) to evaluate its structural stability.

### Molecular dynamics simulation

The iMODS server (//imods.iqf.csic.es/) [32] was employed to conduct normal mode analysis and molecular dynamics simulations of the vaccine–TLR4 complex. These simulations provided insight into the dynamic behavior, structural flexibility, and stability of the docked comp-lex based on eigenvalue distribution and covariance analysis.

## RESULTS

### Sequence retrieval

The AA sequences of LplA and PdhD proteins were retrieved from the UniProt website. The antigenicity of LplA and PdhD proteins was predicted using VaxiJen v2.0. The results showed that both proteins are immunogenic, with antigenic scores of 1.1 and 1.3, respectively, exceeding the threshold value of 0.4. Moreover, allergenicity and toxicity analyses indicated that both proteins are non-toxic and non-allergenic to the selected host species. As mentioned before, antigenicity and toxicity were predicted using the AllerTOP and ToxinPred web servers.

### Epitope mapping

**Figure 1:**
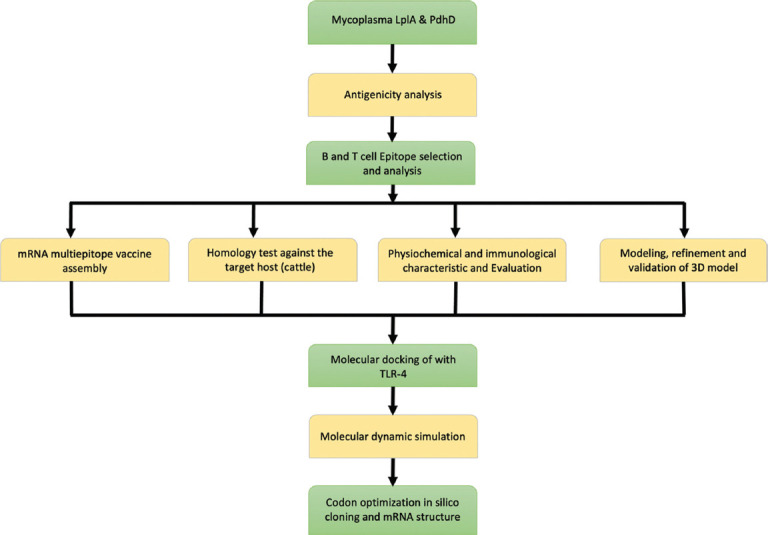
Workflow of the study.

Conserved, antigenic, non-allergenic, and non-toxic T- and B-cell epitopes from LplA and PdhD proteins were selected to construct the proposed multiepitope mRNA vaccine. Based on these criteria, five CTL and six HTL epitopes were selected for inclusion in the vaccine construct, as shown in Tables 1 and 2, respectively. In addition, four linear B-cell epitopes and one conformational B-cell epitope were identified (Table 3). All selected epitopes exhibited antigenic scores above the threshold value of 0.4. These candidate epitopes were subsequently used in vaccine construction and further analyses (Figure 1).

**Table 1:** CTL candidate epitopes that used for constructing of the proposed vaccine.

S. No.	Epitopes	Antigenicity score	Toxicity	Allergenicity	Name of the gene	Conservancy (%)
1	GVCYLIPYK	0.6543	No	None	*LplA*	100
2	ISGDFFAKK	0.8405	No	None	*LplA*	100
3	QMYASMGTK	0.7900	No	None	*PdhD*	100
4	EMAGKAGLK	1.5792	No	None	*PdhD*	100
5	VQTEEGVAK	1.2670	No	None	*PdhD*	100

CTL=Cytotoxic T lymphocyte, *LplA*=Lipoate protein ligase, *PdhD*=Dihydrolipoamide dehydrogenase

**Table 2:** HTL candidate epitopes used to construct the proposed vaccine.

S. No.	Epitopes	Antigenicity score	Toxicity	Allergenicity	Name of the gene	Conservancy (%)
1	FKYLKEHNI	0.4033	No	None	*LplA*	100
2	IKELGAKNV	0.5593	No	None	*LplA*	100
3	FNNISAEEV	0.7647	No	None	LpdA	100
4	WVKMHERKA	0.5443	No	None	*PdhD*	100
5	FVGAREIEV	0.5854	No	None	*PdhD*	100
6	LAAEMAGKA	1.0957	No	None	*PdhD*	100

HTL=Helper T lymphocyte, *LplA*=Lipoate protein ligase, *PdhD*=Dihydrolipoamide dehydrogenase

### Vaccine assembly

**Figure 2:**
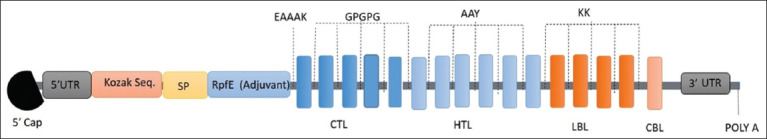
Construction of the modeled vaccine. At the N-terminal Kozak sequence and adjuvant, untranslated regions were added to both ends of the construct. 16 epitopes were used to construct the messenger RNA proposed vaccine, and each epitope was fused with a specific amino acid linker, as shown in graph.

The final structure of the proposed vaccine is illustrated in Figure 2. The proposed vaccine comprises five CTL epitopes linked with GPGPG linkers, six HTL epitopes joined through AAY linkers, and five B-cell epitopes, including four linear ones connected by KK linkers. RpfE was added to the N-terminal as an adjuvant to enhance the antigenicity of the vaccine construct. The HBB and Rabb sequences were incorporated at both ends of the modeled vaccine as untranslated regions. In addition, the Kozak sequence and signal peptide were included to enhance translation and functional expression. A set of immunological tools was used to predict antigenicity, allergenicity, physicochemical properties, structure, and validation of the modeled vaccine.

### Physicochemical analysis of the modeled vaccine

The immunological profile was predicted using the VaxiJen, ToxinPred, and AllerTOP servers. The results (Table 4) indicated that the construct is antigenic (score = 0.835) and non-toxic and non-allergenic to host cells. The physicochemical profile of the vaccine was evaluated using the ProtParam web server. As presented in Table 3, the construct was stable at estimated half-lives of 1.9 h in mammalian cells, 10 h in *E. coli*, and 20 h in yeast. Other properties of the vaccine construct are summarized in Table 4. Overall, the results suggest that the proposed construct is a stable, immunogenic molecule with a favorable half-life in various cellular systems.

**Table 3:** Linear B-cell candidate epitope used for constructing the proposed vaccine.

No.	Start	End	Peptide	Length	Gene	Toxicity
1	6	13	PIRNGEYI	8	*LplA*	None
2	53	62	NPEVEVNFKY	10	*LplA*	None
3	8	10	TKS	3	*PdhD*	None
4	131	156	VVANLEDLKIDY QQSWVKMHERKAKV	26	*PdhD*	None

*LplA*=Lipoate protein ligase, *PdhD*=Dihydrolipoamide dehydrogenase

**Table 4:** Immunogenic and physicochemical characteristics of the vaccine constructs.

Properties of the vaccine construct	Measurement	Note
No. of AA	267	Suitable
MW of the construct	28702	Appropriate
Theoretical pI	9.48	Basic
Formula	C_1286_H_2002_N_358_O_373_S_8_	-
Half-life (*Escherichia coli, in vivo*)	10 h	-
Half-life (mammalian reticulocytes, *in vivo*)	1.9 h	-
Half-life (yeast-cells, *in vivo*)	20 h	-
Grand average of hydropathicity	−0.426	Hydrophilic
Instability index of the proposed vaccine	29.99	Stable
Antigenicity	0.8267	-
Allergenicity	Non-allergic	-
Toxicity	Non-toxic	-

AA=Amino acids, MW=Molecular weight, pI=Isoelectric point

### Prediction of the secondary structure

**Figure 3:**
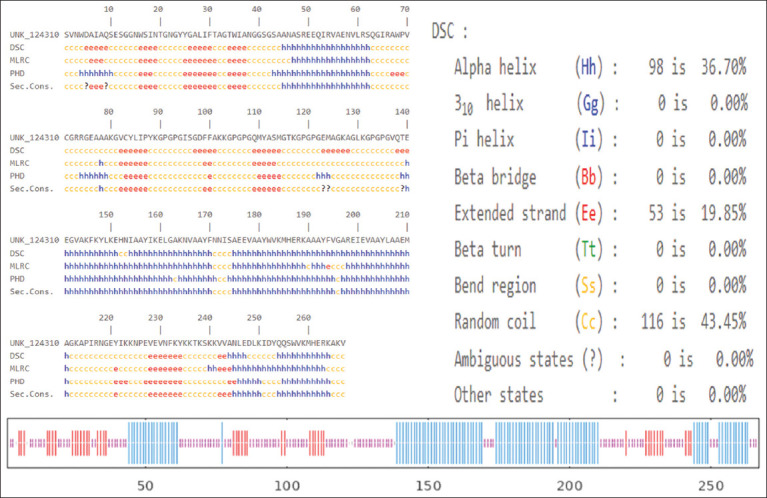
Predicted secondary structure of the vaccine construct. The predicted secondary structure including alpha helix (blue), random coil (orange), and extended stand (red).

As shown in Figure 3, the proposed vaccine consists of 267 AAs, and the presented structures include a random coil, alpha helix, and extended strand. The random coil constituted 43.35% of the total protein structure, whereas the alpha helix and extended strand accounted for 36.70% and 19.85%, respectively. The results revealed that the vaccine construct has a good unfold region that makes it highly recognizable by the antibody of the host.

### Three-dimensional structure

**Figure 4:**
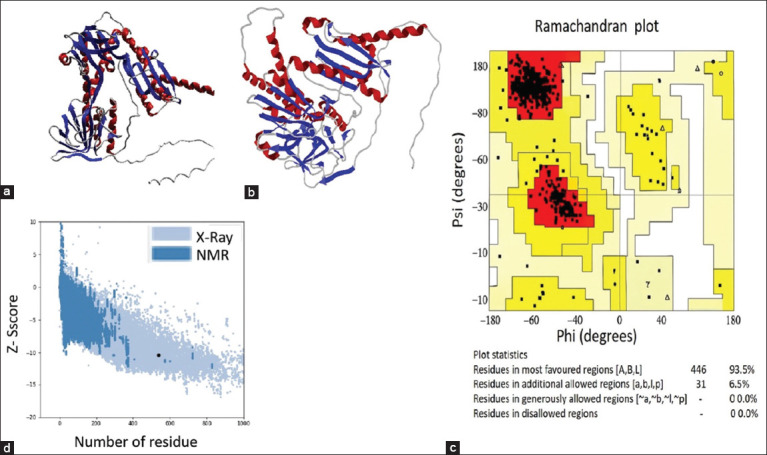
Modeled vaccine structure, refinement, and validity. (a) Three-domination structure of the vaccine construct. (b) Refinement of the 3D structure of the vaccine construct. (c) Analysis of Ramachandran plot and validation of vaccine construct. The Ramachandran plot indicates that 93.5% of residues are situated in the most favored regions, whereas 6.5% of residues are found in the additionally allowed regions. (d) Represents the modeled vaccine Z score.

trRosetta was used to predict the 3D structure of the vaccine. The predicted models relied on a similar structure in the Protein Data Bank (PDB). Model 1 was selected out of ten models based on confidence score (CS score) (Figure 4a). The candidate model was refined with the Galaxy web server. The server predicted five models; among these models, model 1 (Figure 4b) was selected as the refined structure based on its score. The refined structure had the lowest root mean square deviation of 0.414, while the MolProbity score was 1.500. Other structural properties included the Clash score (3.0) and the Rama-favored region (91.0). The validation of the structure was performed using the SAVE web server. The Ramachandran plots show that 93.5% of residues were located in favored areas, whereas 6.5% were located in allowed areas (Figure 4c). ProSA analysis of the construct showed that the Z-score was −10.6. Overall, the estimated construct revealed that the vaccine construct was flexible, stable, and valid (Figure 4d).

### Docking of the modeled vaccine with bovine TLR4

**Figure 5:**
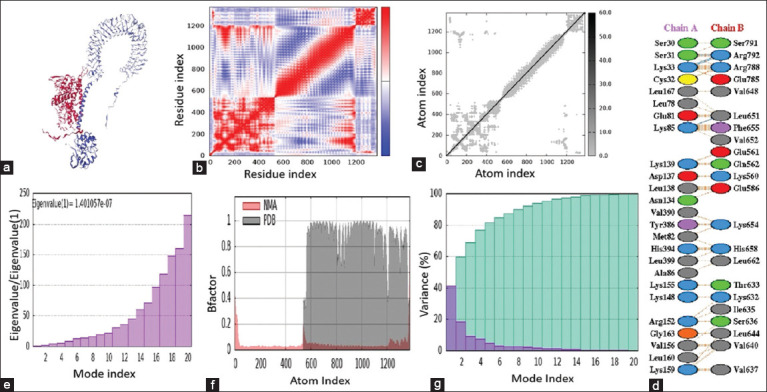
Interaction between the modeled vaccine and bovine toll-like receptors 4 (TLR4). (a) Docking complex of the modeled vaccine with receptor. (b) Graph representing covariance of the complex. (c) Model of elastic network. (d) Model of interaction of amino acids within the docking complex (modeled vaccine and bovine TLR4). (e) B-factor. (f) Eigenvalues value the complex. (g) Individual and accumulative variance of the complex.

Results of docking with the LZerD web server revealed 10 possible models for the interaction between the tested molecules. Based on the model score, model one (Figure 5a) was chosen as a model that represented the possible interaction between the two molecules within the complex. The scores of the selected model included a rank sum score of 213, Generalized Orientation-Dependent All-Atom Statistical Potential score (GOAP) of −203667.64, GOAP rank of 152, Distance-scaled, Finite, Ideal-gas Reference state score (DFIRE) of −129048.92, DFIRE rank of 46, Isothermal Titration score (IT) of −54638.92, and IT rank of 15. Furthermore, the stability of the complex was estimated using the iMODS server. In Figure 5b, the covariance matrix plot shows the degree of dynamic correlation between AAs. Red color refers to correlated AAs, while blue and white colors refer to non-correlated and anti-correlated residues, respectively. In addition, the connection of residues as pairs by spring was represented in the elastic network model (Figure 5c), where harder springs are shown in gray color. Interactions between residues across the interface between the vaccine and bovine TLR4 were predicted using the PDBsum web server. The results show that four hydrogen bonds and 203 non-bonding contacts (Figure 5d) were found across the interface of the two tested molecules. The results represented in Figure 5e, indicate that the complex (modeled vaccine with bovine TLR4) exhibits strong deformability, with eigenvalues of the docked complexes at 1.401057e-7. As shown in Figure 5f, a comparison was performed between network meta-analysis and PDB for the candidate construct. The normal mode and B graph represent the characterization and flexibility of the docking complex. Accumulative and individual variances are represented in Figure 5g, where light green represents cumulative variance while purple color represents individual variation. Overall, the results demonstrate that the complex is strong and stable.

### *In silico* codon optimization and mRNA vaccine structure

**Figure 6:**
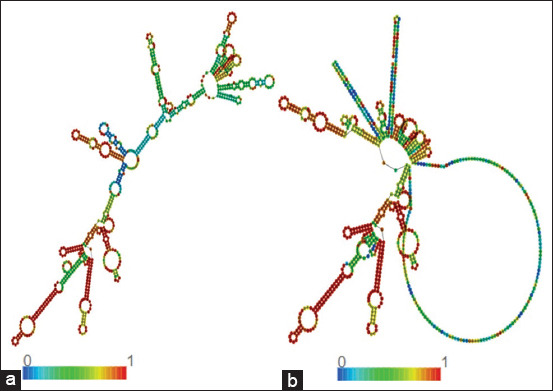
Structure of the messenger RNA (mRNA) construct. (a) Ideal secondary configuration. (b) Central secondary arrangement of the mRNA.

The web server of VectorBuilder (USA) was used to optimize the sequence of the proposed vaccine. The results of optimization show that the GC content of the sequence after optimization was 59.80%, whereas the CAI was recorded as 0.87. The results indicate that translation of the modeled mRNA is possible in cattle (target host). Figure 6 presents the predicted structural arrangement of the modeled RNA molecule with the RNAfold web server. The analysis of the RNA molecule showed that the centroid secondary structure-free energy was −240.10 kcal/mol, whereas it was −321.42 kcal/mol for the thermodynamic ensemble. Overall, the thermodynamic robustness of the modeled RNA was good and stable.

## DISCUSSION

### Clinical impact and control challenges

*M. bovis* is responsible for multiple clinical conditions in cattle, including mastitis, bronchopneumonia, and arthritis, leading to substantial economic losses due to reduced productivity and increased treatment and control costs [9]. The control of *Mycoplasma* infections remains challenging due to the increasing prevalence of antibiotic resistance and the absence of an effective commercial vaccine [33, 34].

### Emerging vaccine technologies

Reverse vaccinology and mRNA-based approaches represent novel vaccine technologies that have been widely deployed against various pathogens during the coronavirus disease-2019 pandemic. Compared to traditional vaccines, reverse vaccinology offers several advantages, including low production costs, robust stimulation of both humoral and cellular immune responses, high specificity, and improved safety [35]. *In silico* methods were used to identify protein epitopes with high antigenicity that is capable of interacting with host immune cells, including B and T lymphocytes.

### Advantages of multiepitope-based design

The use of multiepitope-based vaccines, as opposed to whole-protein formulations, offers multiple advantages, including enhanced immune targeting of conserved regions and reduced risk of adverse sensitization reactions in vaccinated animals [36–41].

### Vaccine construction and target selection

In this study, an mRNA-based subunit vaccine was designed using two *Mycoplasma* proteins (LplA and PdhD) through immunoinformatic tools and molecular modeling. The selected target antigens were LplA and PdhD, both of which play roles in *M. bovis* pathogenicity. Previous studies have documented the roles of these proteins as virulence factors involved in immune evasion and host-pathogen interaction. This is the first study to use LplA and PdhD as target antigens in the design of a multiepitope mRNA vaccine against *M. bovis* infection in cattle.

### Antigenicity and construct features

As anticipated, the selected proteins exhibited high antigenicity scores and were predicted to be non-allergenic in the target host. The final vaccine construct included 16 B-cell and T-cell epitopes, each joined to the next through appropriate AA linkers. As an mRNA-based construct, the vaccine incorporated functional and stability-enhancing sequences, as illustrated in Figure 2.

### Immunogenic potential and safety

Given that subunit vaccines generally exhibit lower antigenicity than whole-pathogen vaccines, an adjuvant (RpfE) was incorporated at the N-terminal to enhance immune stimulation. The results demonstrated that the proposed mRNA vaccine is stable and antigenic, capable of inducing both humoral and cellular immune responses, and exhibits strong binding affinity to host immune receptors. Furthermore, the vaccine candidate is predicted to be safe, with no potential for integration of the mRNA construct into the host genome.

## CONCLUSION

This study successfully employed a comprehensive immunoinformatic and molecular modeling approach to design a novel multiepitope mRNA-based vaccine targeting *M. bovis*, a major pathogen responsible for respiratory, mammary, and reproductive diseases in cattle. Using the conserved, surface-exposed proteins LplA and PdhD as antigenic targets, a total of 16 non-toxic, non-allergenic, and antigenic T- and B-cell epitopes were selected and assembled into a structurally stable vaccine construct. The inclusion of functional elements such as the RpfE adjuvant, untranslated regulatory regions (HBB and Rabb), and signal peptides enhanced the immunogenic profile, translational efficiency, and mRNA stability of the construct.

The predicted vaccine demonstrated strong physicochemical stability (instability index: 29.99; GRAVY: −0.426), high antigenicity (score: 0.835), and robust binding affinity to bovine TLR4 in molecular docking simulations. Structural modeling and validation demonstrated excellent stereochemical quality, with 93.5% of residues falling within the favored regions of the Ramachandran plot and a favorable ProSA Z-score of −10.6. Furthermore, codon optimization yielded a high CAI (0.87) and favorable GC content (59.8%), indicating potential for efficient expression in bovine systems.

The practical implications of this research lie in its potential to guide the development of safe, specific, and cost-effective mRNA vaccines for controlling *M. bovis* infections in cattle, particularly in regions facing increasing antimicrobial resistance. The strength of this study lies in its integrative framework, which combines reverse vaccinology, structural immunology, and transcriptomic stability predictions, providing a holistic preclinical assessment of vaccine viability.

However, limitations include the reliance on *in silico* predictions without experimental validation, which may not fully replicate the complexity of host-pathogen interactions under field conditions. Future scope should include *in vitro* expression analysis, *in vivo* immunogenicity trials in bovine models, and formulation optimization for delivery systems suited to mRNA stability in veterinary applications.

The proposed mRNA-based multiepitope vaccine construct presents a scientifically sound and innovative candidate for combating *M. bovis* infections. With further experimental validation, it holds promise as a viable tool to enhance cattle health, reduce antimicrobial use, and improve productivity in the livestock industry.

## AUTHORS’ CONTRIBUTIONS

DRA: Identified highly antigenic proteins within the *M. bovis* genome. DA and AA: Software and analysis. AA: Drafted and revised the manuscript. All authors have read, reviewed, and approved the final manuscript.
